# Mild features of partial *PAX3* deletion in patients with prenatal Waardenburg syndrome: a case report and literature review

**DOI:** 10.3389/fped.2025.1642132

**Published:** 2025-10-10

**Authors:** Qi Chen, Lin Shi, Yunpeng Chen, Xinyu Cao, Yan Yang

**Affiliations:** ^1^ Genetic and Prenatal Diagnosis Center, Fourth Affiliated Hospital of Jiangsu University, Zhenjiang, China; ^2^ Medical Center for Obstetrics and Gynecology, Fourth Affiliated Hospital of Jiangsu University, Zhenjiang, China; ^3^ Department of Ultrasound, Fourth Affiliated Hospital of Jiangsu University, Zhenjiang, China

**Keywords:** *PAX3*, Waardenburg syndrome, SNP-array analysis, chromosomal aberration, cleft vertebrae

## Abstract

**Background:**

Waardenburg syndrome (WS) is a group of autosomal dominant hereditary disorders characterized by auditory–pigmentary abnormalities. Haploinsufficiency of paired box 3 (*PAX3*) gene is one of the known pathogenic mechanisms. However, clinical phenotypes are difficult to predict precisely in fetuses harboring *PAX3* haploinsufficiency. In this study, we report a family with a *PAX3* deletion encompassing exons 1–4, in which clinical manifestations ranged from normal to mild abnormalities.

**Case presentation:**

A 22-year-old woman (gravida 2, para 0) was referred to our prenatal center at 18 weeks of gestation due to a history of congenital spina bifida in her previous pregnancy. Chromosomal analysis had been performed on fetal tissue from the terminated pregnancy and on amniotic fluid obtained during the current pregnancy. A rare partial deletion of *PAX3* gene was identified and confirmed to be of paternal origin. A diagnosis of WS was established based on the clinical features of the father. However, the newborn from the second pregnancy exhibited normal phenotypes after birth.

**Conclusion:**

This work suggests that deletions encompassing the promoter and functional domains of *PAX3* gene functionally represent a haploinsufficiency mechanism, which leads to variable clinical manifestations. These findings broaden our understanding of copy number variation analysis and genetic counseling in prenatal settings. In addition, *PAX3* gene should be considered a candidate gene in cases presenting with auditory–pigmentary abnormalities or neural tube defects of unknown etiology.

## 1 Introduction

Waardenburg syndrome (WS) is a group of syndromic genetic disorders characterized by auditory–pigmentary abnormalities, primarily inherited in an autosomal dominant pattern (1, 2). It is both clinically and genetically heterogeneous and is usually categorized into four subtypes (WS1–4). Recent studies have identified multiple genetic loci linked with WS, including paired box 3 (*PAX3*), microphthalmia-associated transcription factor (*MITF*), the SRY-box transcription factor 10 (*SOX10*), endothelin 3 (*EDN3*), endothelin receptor type B (*EDNRB*), *KIT* ligand (*KITLG*), and snail family transcriptional repressor 2 (*SNAI2*). These genes encode proteins involved in development and function, and pathogenic variants in them mainly lead to the different clinical types of WS (3–5). Furthermore, growing evidence suggests that *KIT* and *CHD7* genes may act as potential causative factors in WS (6). Specifically, WS1 typical presents with congenital sensorineural hearing loss; pigmentary disturbances in the iris, hair, and skin; and dystopia canthorum (DC) (7). WS2 shares auditory and pigmentary abnormalities of WS1 but lacks DC. WS3 is rarer and more severe, with features similar to WS1 along with musculoskeletal abnormalities (e.g., upper limb malformations). Affected individuals may present with complete heterochromia iridium, partial/segmental heterochromia, hypoplastic or brilliant blue irides, or congenital leukoderma. WS4 is a rare neurocristopathy characterized by Hirschsprung disease and/or other intestinal/neural dysfunctions, in addition to deafness and pigmentary defects (3, 8).

The clinical features of WS include incomplete penetrance and high variability in expressivity, posing challenges for genetic counseling (9). The identification of a heterozygous *PAX3* mutation through molecular genetic testing confirms the diagnosis of WS1 or WS3. However, neither the clinical manifestations nor their severity can be predicted based solely on the presence of pathogenic *PAX3* variants, whether inherited or *de novo*. *PAX3* gene mutations may result in hearing loss, which affects nearly half of patients with WS, whereas *SOX10* mutations may cause more severe clinical consequences (8). WS2 is usually caused by mutations in *MITF*, *SOX10*, or *SNAI2* genes. WS4 follows an autosomal recessive inheritance pattern and is associated with mutations in *EDNRB* or *EDN3*. The genetic causes of WS2 and WS4 cannot be definitively separated. While *SOX10* gene mutations are usually linked to WS4, *SOX10* deletions have also been detected in patients with WS2 (5, 10). This suggests that these genes may constitute a complex regulatory network. Currently, there is no definitive treatment for WS, although life expectancy in affected children is normal (11–13). Morbidity in WS is related to defects in neural crest-derived tissues, including intellectual disability, hearing impairment, ocular defects, skeletal anomalies, and psychiatric disorders (14). Therefore, molecular analysis and genotype–phenotype characterization are critically essential for accurate clinical diagnosis and effective genetic counseling.

In this study, we report a family carrying a partial *PAX3* in structure but exhibiting a haploinsufficiency effect, with variable clinical manifestations among affected individuals. Microarray analysis revealed a heterozygous 521-kb deletion in the 2q36.1 region encompassing exons 1–4 of *PAX3* gene. Our findings underscore the regulatory role of *PAX3* in WS pathogenesis, expand the known clinical spectrum of associated phenotypes, and support the utility of molecular genetic screening in individuals with unexplained auditory–pigmentary abnormalities or neural tube defects (NTDs).

## 2 Samples and methods

### 2.1 Case presentation

**Figure 1:**
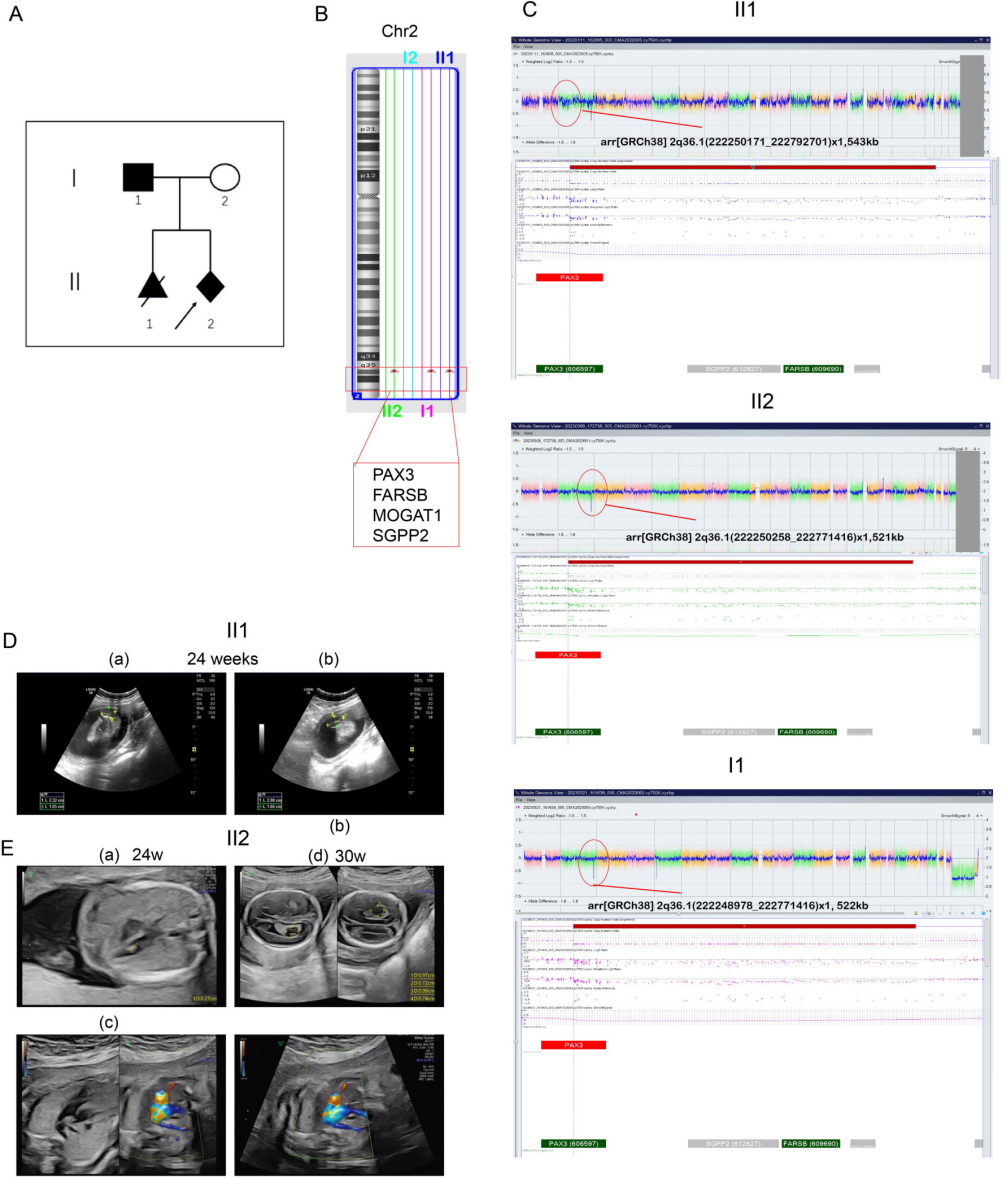
Pedigree and CMA results of the family. (**A**) Family pedigree. The index patient is marked by an arrow. All individuals suspected of having WS1 are shaded. The proband (III2) is indicated by an arrow. III1 was diagnosed with open neural tube defect (ONTD), and the pregnancy was terminated at 20 weeks. II1 carried the same deletion region as III1 and III2. (**B**) CMA result showing a 544-kb deletion of 2q36 in the III1 and proband (III2). Their father also carried the same region of deletion. (**C**) CMA results for II1, II2, I1, and I2. (**D**) Ultrasound at the 24th gestational week revealing cleft vertebrae in II1. (**E**) Ultrasound at the 24th gestational week in the second pregnancy revealing bilateral choroid plexus cysts (a and b), right aberrant subclavian artery (c), and left ventricular bright spot in II2. (d) Ultrasound at the 30th gestational week revealing only the right aberrant subclavian artery in II2.

A 22-year-old woman (gravida 2, para 0) was referred to our prenatal center at 18 weeks of gestation following the termination of her previous pregnancy at 24^+2^ weeks due to congenital spina bifida (Figure 1D). Chromosomal analysis had been performed on the abnormal fetal tissue. In her current (second) pregnancy, a detailed ultrasound examination at 14 weeks of gestation revealed no fetal anomalies (nuchal translucency: 2.0 mm), and all prenatal screening results remained normal throughout gestation. Amniocentesis was performed at 18^+3^ weeks of gestation. Sonography at 24 weeks revealed a bilateral choroid plexus cyst, an aberrant right subclavian artery, and an echogenic intracardiac focus (Figure 1E), with no other structural abnormalities. Follow-up ultrasound examinations showed the persistence of only the aberrant right subclavian artery (Figure 1E-d). After genetic counseling, the patient and her family decided to continue the pregnancy. At 40 + 2 weeks of gestation, she delivered a female neonate (birth weight 3,470 g), with an Apgar score of 10 and an incidental umbilical cord knot.

**Figure 2:**
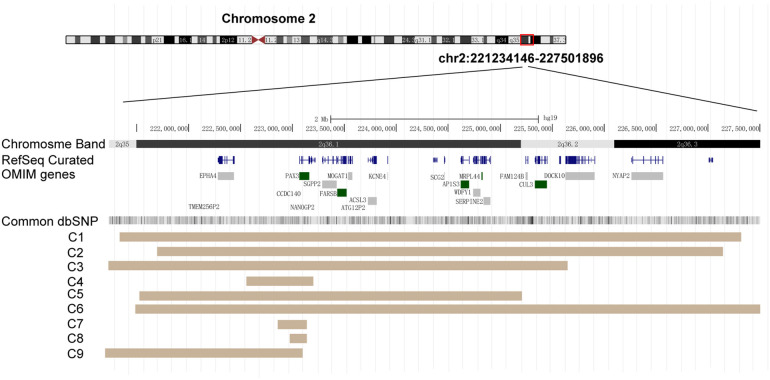
*PAX3* deletion regions in the nine cases. Diagram taken from the UCSC Genome browser [NCBI build 37 (hg19)]. Key genes within this region are displayed. Brown areas represent the covered regions in these cases (C1–C9).

**Figure 3:**
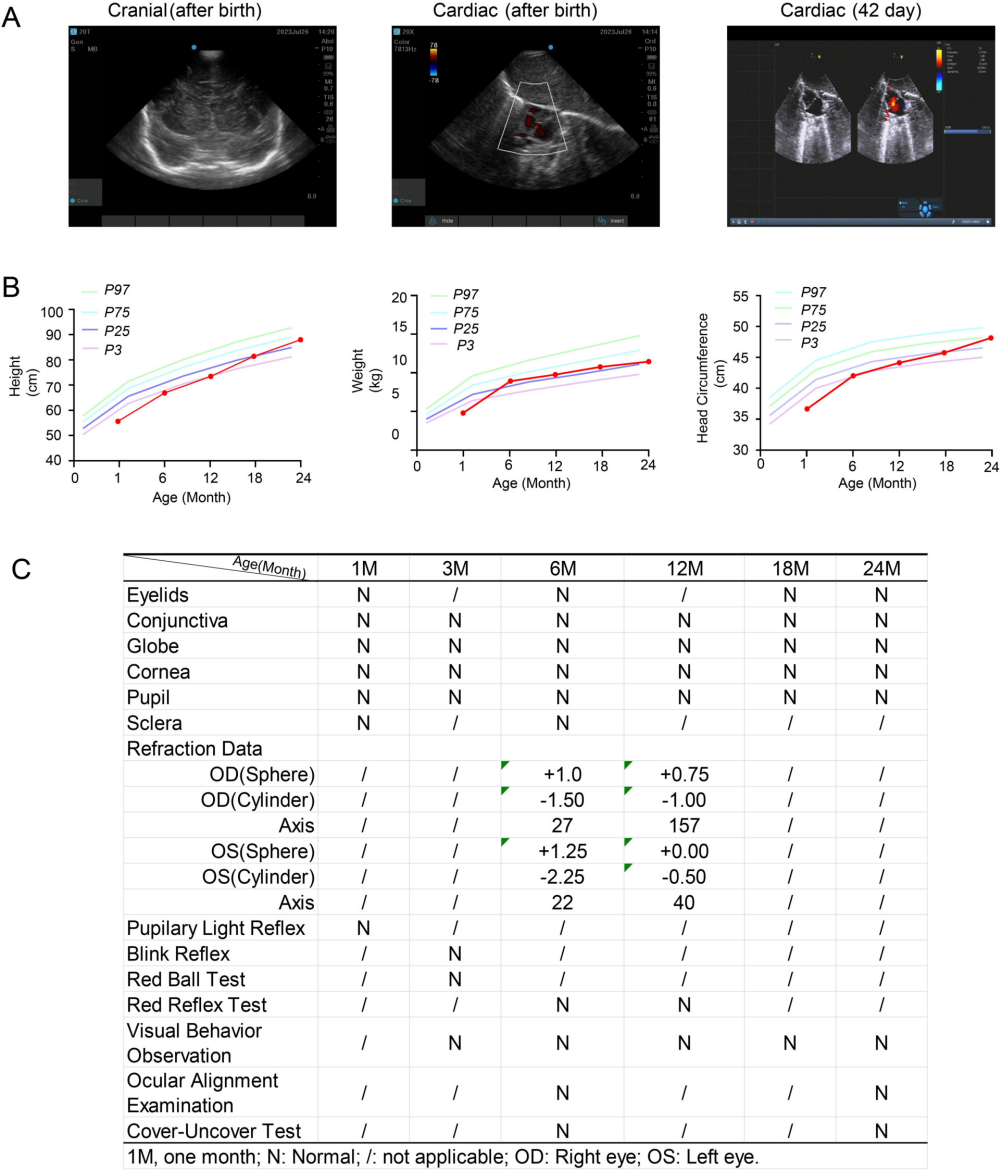
Clinical information of the patient (III2) during routine growth monitoring and developmental surveillance, including anthropometric measurements and vision assessment. (**A**) Cranial and cardiac ultrasounds performed after birth. A follow-up cardiac ultrasound was conducted 42 days after birth. (**B**) Growth parameters, including height, weight, and head circumference, measured at different ages. *P3*, *P25*, *P75*, and *P97* represent the 3rd, 25th, 75th, and 97th percentiles, respectively. (**C**) Visual assessments performed at different ages.

The neonate presented with telecanthus (slightly wide ophthalmic distance) but no cutaneous abnormalities. Postnatal laboratory examinations revealed neonatal infection, as evidenced by abnormal blood tests (total white blood cells: 27.36 × 10^9^/L; serum amyloid A: 20.67 mg/L; central granulocyte ratio: 73%). The infant developed pathological jaundice within 24 h after birth (total bilirubin: 115.7 μmol/L) and exhibited abnormal myocardial enzyme levels. After transfer to the neonatal unit, cranial MRI and chest x-ray were normal, and ultrasound showed no abnormalities in the brain, liver, gallbladder, pancreas, or spleen. Cardiac ultrasound indicated a patent foramen ovale (Figure 3A). At 1 month of age, the infant passed the hearing screening test in both ears, which was conducted using transient-evoked otoacoustic emissions (TEOAE) and automated auditory brainstem response (AABR) testing. The child underwent routine growth monitoring and developmental surveillance, including anthropometric measurements, physical examination, and vision assessments. Growth parameters and developmental milestones were age-appropriate at the time of investigation (Figure 3B). Vision assessments performed at different ages were normal, with no iris abnormalities detected (Figure 3C).

### 2.2 Chromosomal microarray analysis

A total of 250 ng of total genomic DNA was extracted from the amniotic acid, villi, and peripheral blood. The DNA was digested, amplified, and purified. Purified DNA was fragmented, biotin-labeled, and hybridized to the Affymetrix Cytoscan 750K GeneChip. The data were visualized and analyzed using the Chromosome Analysis Suite (ChAS; Affymetrix, USA). All segments were monitored for the degree of overlap with previously identified common copy number variation (CNVs) and evaluated using published literature and several public databases, including DGV (http://dgv.tcag.ca/dgv/app/home), ClinGen (https://www.clinicalgenome.org/), DECIPHER (https://decipher.sanger.ac.uk/), ClinVar (https://www.ncbi.nlm.nih.gov/clinvar/), gnomAD (https://gnomad.broadinstitute.org/), and OMIM (https://www.omim.org/). All reported CNVs were annotated based on the NCBI human genome build 38 (GRCh38). Classification followed the 2020 standards of the American College of Medical Genetics and Genomics (ACMG) and the Clinical Genome Resource (ClinGen) (15). The CNVs were classified as pathogenic (P), variants of uncertain significance (VUS), or benign (B). VUS were further subclassified as likely pathogenic (LP), likely benign (LB), or no subclassification.

## 3 Results

We report a familial case of a 2q36.1 deletion exhibiting clinical phenotypic heterogeneity (Figure 1A). The fetus in the previous pregnancy (II:1) presented with isolated congenital spina bifida without additional anomalies. Chromosomal microarray (CMA) analysis of the fetal tissue identified a 543-kb deletion in the 2q36.1 region [arr(GRCh38) 2q36.1(222,250,171_222,792,701) × 1]. In the current pregnancy, amniocentesis (II:2) revealed the same deleted region at 2q36.1 [arr(GRCh38) 2q36.1(222,250,258_222,771,416) × 1] (Figures 1B,C). CMA analysis of the origin verified that the deletion originated from the father [I:1, arr(GRCh38) 2q36.1(222,248,978_222,771,416) × 1, 522 kb] (Figures 1A,C). The father (I:1) displayed characteristic WS facial features (DC, synophrys, a white forelock, and a broad nasal root) but had normal hearing, intellectual ability, and growth. Notably, the newborn (II:2) exhibited atypical clinical phenotypes, presenting with DC and abnormal metabolic disturbances but without major structural anomalies. In this family with inherited CNVs, the deleted genomic region encompassed *PAX3* (OMIM 606597), *FARSB* (OMIM 609690), *MOGAT1* (OMIM 610268), and *SGPP2* (OMIM 612827) genes in the 2q36 region (Figure 2). This region included the 5′ upstream region and the first four exons of *PAX3* gene, which demonstrated sufficient evidence for haploinsufficiency (HI score: 3). Based on ACMG guidelines (15), this region was classified as likely pathogenic (1A + 2C-1, score: 0.9). Hence, we concluded that this deletion including the promoter and exons 1–4 of *PAX3* gene represents the genetic pathology of this family, which exhibited clinical phenotypes of variable severity.

## 4 Discussion

In this study, three affected family members carried identical 2q36.1 deletions but exhibited variable manifestations, establishing an inherited pattern of atypical WS. The deleted region encompassed four OMIM genes, including *PAX3* (606597), *SGPP2* (612827), *FARSB* (609690), and *MOGAT1* (610268). To date, there is little evidence linking deletions of *SGPP2*, *FARSB*, and *MOGAT1* gene to specific clinical phenotypes. Existing literature suggests that *SGPP2* gene may function as a novel vitamin D-responsive gene associated with lung function (16) and may serve as a potential immune regulator in inflammatory bowel disease (IBD) (17). Biallelic *FARSB* mutations are known to cause loss-of-function effects and result in phenylalanine-tRNA synthetase (PheRS)-related recessive disorders manifesting as significant growth restriction, brain calcification, and interstitial lung disease (18, 19). MOGAT1 has been implicated in glucose metabolism regulation in mouse models (20). In contrast, *PAX3* gene is involved in the development of cardiac tissue, melanocytes, and enteric ganglia, as well as the formation of the central nervous system, somites, and skeletal muscle. Pathogenic *PAX3* deletions are known to cause embryonic NC dysplasia and can lead to craniofacial-deafness-hand syndrome (CHDS) (9), underscoring its critical developmental functions and its alignment with the observed phenotypic spectrum observed in this family.

To date, *PAX3* mutations have been widely identified in patients with WS1 or WS3, including missense or non-sense variants identified by whole-exome sequencing (WES), as well as CNVs detected by CMA analysis (8). However, studies focusing on a single *PAX3* deletion identified using CNV analysis remains limited. Several studies have reported larger chromosomal deletions in the 2q36 region, including additional key genes besides *PAX3*. For example, prenatal genetic testing has identified a *de novo* 5.6-Mb deletion at 2q36.1q36.3 (containing 17 coding genes, including the *PAX3* and *EPHA4*). The fetus exhibited syndromic manifestations, including craniofacial dysmorphism with typical hypertelorism, flat facial profile, moderate microretrognathia, upward and backward slanted ears, broad forehead, large and flat occiput, cleft palate, microcephaly, and lumbosacral spina bifida (14). Notably, the size of the deleted segments does not appear to correlate with the expressivity of clinical phenotypes. For example, two small unrelated boys shared nearly the same deletion in the 2q36 region as in the above case. However, their clinical outcomes differed markedly: one presented with severe bilateral hearing loss, short stature, and intellectual disability, but without white forelocks, heterochromia of the irides, skin depigmentation, cardiac and renal malformations, or vertebral anomalies (21), whereas the other exhibited facial dysmorphism and severe short stature, but had normal intelligence quotient and hearing, with no evidence of heterochromia (22). Furthermore, a 16-year-old girl exhibited typical WS features, including congenital hearing loss, brilliant blue irides, DC, shortened upslanting palpebral fissures, and hypoplastic alae nasi. Single nucleotide polymorphism (SNP) analysis revealed a *de novo* 862-kb deletion in the 2q36.1 region, containing only *PAX3*, *CCSC140*, and a portion of *SGPP2* gene (23). Thus, the clinical characteristic dysfunction is mediated predominantly by *PAX3* gene haploinsufficiency, independent of the deletion size.

Moreover, inherited CNVs involving *PAX3* gene have a low incidence, but the clinical manifestations of each affected member are more complex and highly variable. A single affected family can display a variable WS1 phenotype, with only the proband exhibiting severe hearing loss in the right ear and the other family members displaying only mild facial dysmorphism (24). In one reported family, an inherited 2q36 deletion involving the whole *PAX3* gene was validated in five members. Among them, three displayed DC and two had a white hair patch. The father and grandfather of the proband exhibited only abnormal nasolacrimal ducts. None of the affected individuals has hearing problems or heterochromia of the iris (25). Thus, these observations provide clear evidence that *PAX3* deficiency leads to WS, but the detailed phenotypes show incomplete penetrance and marked variability, especially in cases of inherited deletions (Table 1, Figure 2).

**Table 1:** Comparison of deletion size and clinical phenotypes in patients with PAX3 deletions at 2q36.

Reference	Case	Sex/age	Position	Extent (kb)	Protein-coding genes	WS-related feature	Inheritance	Other phenotypes
Goumy et al. (14)	C1	F/prenatal	Arr(GRCh37)2q36.1q36.3 (221,835,372_227,501,896) × 1	5.6 Mb	*EPHA4*, *PAX3*, *CCDC140*, *SGPP2*, *FARSB*, *MOGAT1*, *ACSL3*, *KCNE4*, *SCG2*, *AP1S3*, *WDFY1*, *MRPL44*, *SERPINE2*, *FAM124B*, and *CUL3*	Craniofacial dysmorphism (hypertelorism, flat face, moderate microretrognatism, upward and backward slanted ears, broad forehead, large and flat occiput, cleft palate, microcephaly), and lumbosacral spina bifida	dn	—
Guan et al. (21)	C2	M/6	Arr(GRCh37)2q36.1q36.3 (2:222,119,829_227,294,482) × 1	5.175 Mb	*PAX3*, *EPHA4*, *ACSL3*, *AP1S3*, *CCDC195*, *CUL3*, *DOCK10*, *FAM124B*, *FARSB*, *KCNE4*, *MOGAT1*, *MRPL44*, *NYAP2*, *RNF228*, *SCG2*, *SERPINE2*, *SGPP2*, and *WDFY1*	Severe syndromic bilateral sensorineural deafness, flat facial profile, and ocular hypertelorism	dn	Severe decay teeth, slight ulnar deviation of the hands, single transverse palmar crease, short stature, and intellectual disability
Li et al. (22)	C3	M/4 years and 6 months	Arr(GRCh37)2q35q36.2 (221,234,146_225,697,363) × 1	4.46 Mb	*PAX3*, *EPHA4*, *CUL3*, *DOCK10*, *SERPINE2*, *FARSB*, *SGPP2*, *ASCL3*, *MRPL44*, *WDFY1*, *AP1S3*, *KCNE4*, *FAM124B*, *MOGAT1*, and *SCG2*	DC, mild synophrys, slightly upward slanted palpebral fissure, posteriorly rotated ears, alae nasi hypoplasia, and micrognathia	dn	Severe short stature
Drozniewska and Haus al. (23)	C4	F/16	Arr(GRCh37)2q36.1 (222,562,885_223,424,791) × 1	862 kb	*PAX3*, *CCDC140*, and part of *SGPP2*	Congenital hearing loss, brilliant blue irides, DC, shortened upslanting palpebral fissures, and hypoplastic alae nasi	dn	Hirsutism, hypercholesterolemia, seborrheic dermatitis, and astigmatism
Matsunaga et al. (24)	C5	Female/4 years and 4 months	rsa 2q36(PAX3,MITF, SOX10) × 1	—	*PAX3,* and 17 other genes	Severe hearing loss (right ear), DC, medial eyebrow flare, and a white forelock	mat	Ketotic hypoglycemia
		Female/NR				Heterochromia iridis, DC, and medial eyebrow flare	mat	—
		Female/NR				Early graying (around 20 years old), DC, and medial eyebrow flare	pat	—
		Male/NR				DC, medial eyebrow flare	—	—
Macaskill et al. (25)	C6	M/1	2q36 region	—	*PAX3*	A hair white patch and hypertelorism, DC	pat	—
		M/NR				Abnormal nasolacrimal ducts	pat	
		M/NR				Nasolacrimal stenosis	—	
		M/NR				Gray hair and DC	pat	
Yang et al. (13)	C7	F/2	rsa 2q36 (PAX3) × 1	—	*PAX3* (1–8 exons)	DC, a white forelock, bilateral brilliant blue irides, and bilateral hearing loss	pat	Thumb polydactyly (left hand)
Jin et al. (12)	C8	F/9	Arr(GRCh37)2q36.1 (223,153,899_223,164,405) × 1	10.26 kb	*PAX3* (1–4 exons) and *CCDC140*	Mild cutaneous syndactyly, DC, and faint synophrys	pat	Bilateral finger contractures
		M/NR				Sensorineural deafness, DC, bright blue eyes (bilateral), synophrys, a white forelock, and a broad nasal root	pat	—
		M/NR				DC, bright blue eyes (left), synophrys, a white forelock, and a broad nasal root	NR	Arthrogryposis of the bilateral fifth fingers
Borg et al. (11)	C9	M/11	ish del(2)(q35)	4.23–4.41 Mb	*PAX3* (promoter and 5′ untranslated region)	Upward slanting palpebral fissures, mild DC, and prominent antihelices	dn	Intrauterine growth retardation, hydrocephalus, developmental delay, and autism

DC, dystopia canthorum; dn, *de novo*; F, female; M, male; mat, maternal; NR, not reported; pat, paternal.

For each patient, the corresponding references are reported. The deletions are described with the position of the first and last abnormal probe (GRCh37/hg19).

Deletions of *PAX3* that encompass both its promoter and exons are rarely reported in patients with WS (Table 1). To our knowledge, exons 1–4 encode two key domains of the *PAX3* protein, including the paired box domain (PD) and the homeodomain (HD), both specific for DNA-binding activity (26). Pathogenic variants or deletions affecting these two domains alter the transcriptional regulation of downstream targets of *PAX3*, leading to functional abnormalities. In our case, the deleted region encompassed the 5′ upstream region besides the first four exons of *PAX3*. This deletion is expected to result in a complete loss of gene products, representing a haploinsufficiency variant; despite this, the clinical expressions exhibited phenotypic heterogeneity. For instance, an inherited *PAX3* deletion including exons 1–8 was annotated as likely pathogenic, yet not all clinical features of WS were observed in affected individuals. DC was the most common phenotype in all patients, whereas other features, such as pigmentary abnormalities of the iris, hair, or skin, were present in some individuals. Only one of nine affected individuals exhibited synophrys or hearing loss (13). Another affected family carried an inherited 2q36.1 deletion of 10.26 kb, containing exons 1–4 of *PAX3* gene. Although the deletion was small, the individuals displayed distinct WS-related phenotypes. In addition to having DC and synophrys, the proband had bilateral contractures of all 10 fingers, and her father and brother shared similar facial features, including a white forelock and a broad nasal root. In addition, her father had arthrogryposis of the bilateral fifth fingers, while her brother presented with sensorineural deafness (12). Notably, the exon deletions in *PAX3* gene are consistence with those in our case, although the clinical manifestations differ. In addition, deletion of *PAX3,* including the promoter and 5′ untranslated region, has been related to mild WS, including DC, in a boy with a *de novo* 2;8 translocation (11). Therefore, these observations suggest that the larger size of the deleted region and key genes, such as *PAX3* and *EPHA4*, act as pathogenic drivers of clinical phenotypes .Overall, patients with deletions of varying sizes in *PAX3* gene—particularly those involving key regions such as the 5′ upstream region and functional exons—exhibit WS features. However, affected family members may still show phenotypic variability due to incomplete penetrance or the influence of secondary genetic or environmental factors. In accordance with ACMG guidelines and supported by previous literature, the partial deletion of *PAX3* in our case is considered the key genetic driver of WS, based on the observed clinical phenotypes.

Studies have demonstrated that families with inherited *PAX3* mutations display multiple symptoms of WS in various combinations. A study described a fetus with severe spina bifida, including myelomeningocele and Arnold–Chiari malformation, which was caused by a missense variant in *PAX3* (c.124G > C.pGly42Arg). The family history displayed notable WS features, with deafness in the left ear, DC, and white forelocks. The father of the proband exhibited only heterochromic irides. The proband received a ventriculoperitoneal shunt at birth to treat hydrocephalus (27). Another WS family with a splice-site mutation in *PAX3* exhibited no congenital hearing loss. In this family, only one fetus presented with severe spina bifida and facial dysmorphisms, while other affected individuals presented with DC, heterochromia iridis, and/or synophrys (28). Furthermore, *in vivo* analyses of *PAX3* gene functions revealed its important role in sacral neural crest development and associated disorders in humans. In detail, lineage-specific deletion of *PAX3* by G protein-coupled receptor 161 (Gpr161) knockdown in mice led to cranial vault and facial bone hypoplasia, vertebral abnormalities, and the closed form of spina bifida during embryonic development, likely mediated by decreased Wnt/β-catenin signaling (29). Another study in mice provided evidence that *PAX3* haploinsufficiency is a likely risk factor for the pathogenesis of congenital hydrocephalus (30). These findings suggest that genetic variations in *PAX3* play an essential role in WS phenotypes, but any divergence in effects of different *PAX3* variants is largely overshadowed by multifactorial elements within the genetic background of the patient. In this study, the fetus from the first pregnancy exhibited cleft vertebrae on mid-gestation ultrasound, without other structural abnormalities, and the pregnancy was subsequently terminated. Although the fetus carried the same *PAX3* deletion, further phenotypic details were unavailable to confirm a definite diagnosis of WS. To our knowledge, NTDs are multifactorial and involve both environmental and genetic factors. Environmental factors include primarily folate deficiency, maternal obesity, diabetes, and exposure to teratogens. Genetic factors are more complex, including chromosomal syndromes, like trisomy 13, trisomy 18, and certain CNVs (31), and pathogenic variants in genes such as *Vinculin* (32), *PDGFRA*, *MAT1A*, and other missense variants (33). In other words, human NTDs are thought to arise from interactions among multiple gene variants and genetic modifiers. The genetic variants associated with NTDs are often polygenic, rather than resulting from simple inheritance of single-gene mutations (34). Furthermore, several studies have reported the occurrence of NTDs in patients with *PAX3* mutations (2, 27, 35). This association is primarily because *PAX3* gene encodes a developmental TF expressed in neural crest cells of the spinal ganglia, craniofacial mesectoderm, and limb mesenchyme during embryogenesis (36). In addition, *PAX3* plays important roles in the migration and differentiation of melanocytes, which also originate from the embryonic neural crest. *PAX3* haploinsufficiency has been associated with neural tube defects in mice (29, 36), although reports in humans remain limited (14, 27). Nevertheless, the severity of spina bifida associated with *PAX3* deletion warrants further investigation.

In summary, this study reveals that deletions affecting the promoter and functional domains of *PAX3* gene functionally act as haploinsufficiency variants, disrupting its transcriptional regulation and leading to clinical manifestations. Although a NTD was observed in one case, strong evidence directly linking *PAX3* to NTDs is lacking. Notably, the phenotypic spectrum of WS is highly variable, even among individuals carrying similar deletions. In our study, except for the case with an NTD, the other familial cases exhibited mild phenotypes, highlighting the incomplete genotype–phenotype correlation in WS. Thus, genetic testing should not be the sole determinant in clinical decision-making; it must be integrated with detailed clinical assessments to guide effective management.

## Data Availability

The original data and figures in the study are included in the article; further inquiries can be directed to the corresponding author.
